# Exploring Pakistani Physicians' Knowledge and Practices Regarding High Alert Medications: Findings and Implications

**DOI:** 10.3389/fphar.2022.744038

**Published:** 2022-03-10

**Authors:** Zia Ul Mustafa, Shahzaib Haroon, Naeem Aslam, Ahsan Saeed, Muhammad Salman, Khezar Hayat, Naureen Shehzadi, Khalid Hussain, Amer Hayat Khan

**Affiliations:** ^1^ Discipline of Clinical Pharmacy, School of Pharmaceutical Sciences, Universiti Sains Malaysia, George Town, Malaysia; ^2^ Department of Pharmacy Services, District Headquarter (DHQ) Hospital, Pakpattan, Pakistan; ^3^ Department of Medicine, Faisalabad Medical University, Faisalabad, Pakistan; ^4^ Department of Surgery and Allied, District Headquarter Hospital (DHQ), Pakpattan, Pakistan; ^5^ Department of Surgery and Allied, DHQ Teaching Hospital, Sahiwal, Pakistan; ^6^ Department of Pharmacy, The University of Lahore, Lahore, Pakistan; ^7^ Institute of Pharmaceutical Sciences, University of Veterinary and AnimalSciences, Lahore, Pakistan; ^8^ College of Pharmacy, Punjab University, Lahore, Pakistan

**Keywords:** medication, physicians, errors, awareness, administration, regulation

## Abstract

**Introduction:** While many low-middle income countries (LMICs), including Pakistan, try and ensure patient safety within available resources, there are considerable concerns with medication use. Unsafe and inappropriate medication use, especially high alert medications (HAMs), is one of the important factors compromising patient safety and quality of care. Besides economic loss, HAMs contribute to greater morbidity, hospitalization, and mortality. Physicians as key members of the provision of healthcare are expected to be well aware of the administration and regulations surrounding HAMs. However, the current status is unknown in Pakistan. Consequently, the objectives of this study were to evaluate the knowledge of Pakistani physicians about the administration, regulation, and practices related to HAMs. This builds on our recently published study with nurses.

**Methods:** An online cross-sectional study design was used, and data were gathered from the physicians throughout Pakistan using previously used self-administered questionnaires during a period of 5 months (January 1 to May 30, 2021). All data were entered and analyzed using SPSS 22 for Windows.

**Results:** Physicians (847) who provided consent were enrolled in the study. Most physicians (62.2%) were male, aged between 25 and 30 years (75.2%) and had 2- to 5-year work experience (50.9%). About 27% were working in the emergency departments. The median (IQR) knowledge score for HAMs administration and regulation was 5 (3) and 5 (2), respectively. About 46.4% of respondents were found to have moderate knowledge about HAMs; increasing age, work experience, and higher qualifications were significantly associated (*p* < 0.05) with better HAMs knowledge. Around 58% had good practices relating to HAMs during their routine work. Median practice scores increased significantly (*p* < 0.05) with age, work experience, and postgraduate qualification.

**Conclusion:** Most Pakistani physicians possess moderate knowledge about HAMs administration and regulations. However, their practices relating to the HAMs administration and regulations are typically sub-optimal. Consequently, HAMs awareness needs to be improved by including course content in the current curriculum, provision of hospital-based continuous training programs about patient safety and care, and establishment of multi-disciplinary health care teams, including board-certified pharmacists and specialized nurses, for the effective execution of medication use process in Pakistani hospitals in the future.

## 1 Introduction

Medication is one of the frequently used modalities to treat illnesses throughout the world. However, improper medication use has a catastrophic impact on patients increasing adverse events (AEs), morbidity, prolonged hospital stays, costs, and mortality (Al Hamid et al., 2014; Aderemi-Williams et al., 2015; Watanabe et al., 2018; Formica et al., 2018; Giardina et al., 2018) Patient safety is one of the fundamental patient rights and should be ensured during the health care provision process.

According to the World Health Organization (WHO), AEs are included in the top 10 leading causes of death and disability worldwide (WHO, 2019a). Overall, 10% of hospital admissions are reported to be due to AEs following medication (WHO, 2015). More recently among countries in the Organization for Economic Co-operation and Development (OECD), it has been estimated that 15% of total hospital expenditure resulted from AEs (Slawomirski et al., 2017). In addition, in high-income countries, one out of every 10 hospitalized patients were harmed with AEs, with half of them considered preventable (WHO, 2019a). In the United States, more than 7 million hospitalized patients are affected by medication errors (MEs), and MEs are estimated overall to cost $40 billion annually (Tariq et al., 2020). In the United Kingdom, 2.37 million MEs are reported with an economic burden of £98.5 million per year (Hodkinson et al., 2020). The situation is even worse in low-middle income countries (LMICs) where more than 134 million AEs are reported due to unsafe medication, with more than 2.6 million deaths every year (WHO, 2019a). Patient safety is a key concern in the Eastern Mediterranean region where 18% of hospitalizations are associated with AEs; 3% lead to permanent disability or deaths with more than 80% AEs considered preventable (WHO, 2015).

Approximately two decades ago, the WHO instigated activities to enhance patient safety and proposed active debate in World Health Assembly (WHA). The subsequent resolution on patient safety urged Member States to take initiatives against patient harm. This campaign led to the development of safe medication programs across the world (WHO, 2019b). In 2021, again, a resolution presented in the 72nd WHA demonstrated the need to improve patient safety by implementing continuous training and skill development among health care professionals (WHO, 2021).

High alert medications (HAMs) are a special class of medicines bearing a greater risk of patient harm than other medicines when prescribed or administered inappropriately (Labib et al., 2018; Salman et al., 2020). HAMs include chemotherapeutic agents, cardiovascular medicines, anticoagulants, opioids and their derivatives, insulin, neuromuscular blocking agents, benzodiazepines, and certain electrolytes in high concentrations (Zyoud et al., 2019). Consequently, prescription, administration, storage, and handling of HAMs should require specific protocols and regulations to reduce or prevent harm related to their prescribing. The prevalence of MEs due to HAMs ranges from 3.8% to 100% in hospital settings with a pooled prevalence rate of 16.3% (Alves et al., 2021). The severity of these errors in this systematic review ranged from 0.1% to 19.2% for moderate errors and 0.2% to 15.4% for serious errors, and 1.9% lead to death (Alves et al., 2021). MEs related to HAMs can occur due to multiple factors including poor communications between health professionals and patients, the improper passage of medication information, organizational factors, interruption during the medication process, psychological impairment of staff, overburden on healthcare professionals (HCPs), lack of regulation and policies, and insufficient awareness among HCPs (Keers et al., 2013; Sessions et al., 2019).

Pakistan is a LMIC located in South Asia with a struggling health care delivery system due to poor governance, inconsistent health policies, and insufficient resources (Kurji et al., 2016). Despite this, there have been a number of initiatives facilitated by the Government and others in Pakistan to enhance patient safety (Jafree et al., 2017); however, patient safety is challenging. It is recently reported that more than half a million deaths occur in Pakistan every year due to MEs, substantially higher than in the United States where the number of causalities due to MEs range between 7,000 and 9,000 per year (Dawn newspaper, 2017; Tariq et al., 2020). A previous study conducted by Mahmoud et al. among low-middle income Asian countries including Pakistan revealed that more than 95% of MEs were prescribing errors (Mahmoud et al., 2020).

A previous study undertaken in the United States indicated that most of the HCPs did not obtain sufficient training on HAMs during their education regarding medication use and HAMs. More specifically, the involvement of prescribers in the training on HAMs was reported to be lower among physicians compared with pharmacy and nursing personnel (Engels and Ciarkowski, 2015). Being an important member of multidisciplinary health care provision teams, physicians need to be informed about all aspects regarding medicines including their administration and potential harm.

Overall, physicians are a key player in addressing issues relating to patient safety as they are recognized as often initiators of the medication use process across sectors after examining the patients according to their needs. In addition, they often need to play the role of an expert in prescribing the right drug for the right patient with the right dose via the right route of administration (Maxwell, 2016). While there are multiple factors that contribute to MEs (Mutair et al., 2021), these are reduced in high-risk situations if they are properly educated, trained, and equipped to prevent such medicine-related harms (Wittich et al., 2014). Consequently, their knowledge regarding administration, regulations, and practices related to HAMs is of considerable importance to prevent HAMs-related errors among patients. Recently, the death of a 9-month-old baby girl due to abrupt administration of 15% potassium chloride (KCl) enhanced issues of HAMs errors in Pakistan (Salman et al., 2020). In view of this, the objectives of this study were to evaluate the awareness of Pakistani physicians about HAMs administration, regulations, and practices during their routine work. We have previously reported on HAM knowledge among nurses (Salman et al., 2020), and we are aware of previous publications regarding concerns with medication safety among physicians and pharmacists in Pakistan (Jafree et al., 2017; Mahmoud et al., 2020). We wanted to build on this to provide future guidance.

## 2 Methods

### 2.1 Study Design and Setting

An online cross-sectional, questionnaire-based survey was undertaken among the registered medical practitioners of public sectors and primary, secondary, and tertiary hospitals of all provinces [Punjab, Sindh, Khyber Pakhtunkhwa (KPK), and Baluchistan] and the capital territory (Islamabad) of Pakistan.

### 2.2 Inclusion and Exclusion Criteria

All physicians registered with the Pakistan Medical Commission (PMC) (2020) currently serving in Pakistani public hospitals and voluntarily willing to participate in this survey were included in our study. Participants not registered with the PMC, medical students currently out of practice, and those not willing to participate due to any reason were excluded from the current survey.

### 2.3 Sample Size Calculation and Data Collection

According to the PMC, 281,072 physicians were registered throughout Pakistan (PMC, 2020). The sample size of the current study was calculated from an online sample size calculator, Rao soft, by considering 50% response distribution, 5% margin of error, and 95% confidence interval. A minimum of 384 physicians were required in the study. However, keeping in view the limitations of convenience sampling and web-based survey design, we empowered the sample by including a design effect. According to earlier studies (Wejnert and Heckathorn, 2008; Masoud et al., 2021), the minimal adequate design effect for convenience-sampled studies is 2. Hence, an adjusted minimum sample of 768 physicians was needed for this study.

A convenient sampling technique was subsequently employed by the investigators, and all potential participants were provided with a link to the e-questionnaire (Google Form) through WhatsApp, Gmail, and Facebook messenger groups of doctors, with a request to complete the survey. Online informed consents were taken from all the study participants prior to their enrolment in the current survey. The link was re-shared after a few days to remind non-participants to submit their responses.

### 2.4 Study Tool

The study tool used in this survey was adapted from a previous study (Hsaio et al., 2010) after permission from the concerned. As the original study questionnaire was in English and the curriculum taught to all physicians in Pakistan is also in English (Salman et al., 2020b), there was no need to translate the study tool into the native language (Urdu). The same data collection tool was employed in a previous study conducted among the registered nurses of Pakistan to extract information about their awareness to HAMs-related administration and regulations (Salman et al., 2020). Moreover, a pilot study was conducted among 20 physicians to improve the utility of study tool for the current study to enhance its robustness. During the pilot study, all the participants were asked about the clarity, understandability, and relevance of all the questions and response options of the study instrument. The original English questionnaire from Hsaio et al. had 20 items to evaluate knowledge about HAMs. However, the questionnaire used in the present study had 19 items because one item (Item # 10, related to Port-a-Cath) was excluded based on the findings of the pilot study to determine content validity. The content validity index after removing the aforementioned item reached 1. Alongside this, Cronbach's alpha of the questionnaire was found to be 0.814, showing adequate internal consistency. We also revised the instrument based on the suggestions of the participants (pilot study) to improve the clarity and understandability of questions. The final content of the study tool had the following four sections:

Section 1 contained six items about the demographic characteristics of the study participants like age (<25 years, 25–30 years, 31–35 years, and ≥36 years), gender (male or female), level of education (graduate or post-graduate), category of the hospital where they were currently serving (primary, secondary, or tertiary care hospital), years of experience (<1 year, 1–5 years, and >5 years), and working department [intensive care unit (ICU), critical care unit (CCU), emergency, surgery etc.].

Section 2 contained nine questions related to the knowledge about HAMs administration. Participants were requested to choose one response from three given options, i.e., yes, no, and do not know.

Section 3 comprised 10 questions about the HAMs-related regulations. This section also had three different options for the participants to select from.

Section 4 had five questions related to the practices regarding HAMs including one question reverse coded to avoid cognitive bias. Participants were allowed to select one option from the five-item Likert scale including always, often, sometimes, rarely, and never.

### 2.5 Calculation of the Scores

The scoring system employed in the current study built on the previous study among registered nurses (Salman et al., 2020). Each right response from Sections 2 and 3 was given a score of 1, while a wrong or do not know response was scored 0. Consequently, the total knowledge score on HAMs administration and regulation were 9 and 10, respectively. To calculate the overall knowledge score, each correct answer in Sections 2 and 3 (total of 19 questions) was given 5 points, with a total score of 95; incorrect or do not know answers were scored 0. Knowledge scores were classified as good knowledge ≥70%, moderate knowledge 50–69%, and poor knowledge <50%. The total score related to HAMs practices ranged 5–25; with ≥70% scores indicating good practices, 50–69% as moderate, and <50% scores as poor practices.

### 2.6 Ethical Approval

Ethical approval of the current study was obtained from the Ethics Committee, Department of Pharmacy Practice, the University of Lahore (UOL) vide approval number REC/DPP/FOP/29 before initiation of study (December 28, 2020). Moreover, online informed consent was obtained from all survey participants before their enrolment in the study. Any queries related to the execution and purpose of this research were sorted out by the investigators. Participants who decided to leave this survey during response filling could do it without any restriction.

### 2.7 Statistical Analysis

The categorical data were presented as frequency and percentages. Median and interquartile ranges (IQRs) were expressed for continuous variables. For inferential analysis, Kruskal–Wallis and Mann–Whitney tests were performed for continuous data. All data analyses were carried out using the Statistical Package for the Social Sciences (SPSS Inc., version 22, IBM, Chicago, IL, United States). A *p* < 0.05 was set as statistical significance.

## 3 Results

### 3.1 Demographic Characteristics

**FIGURE 1:**
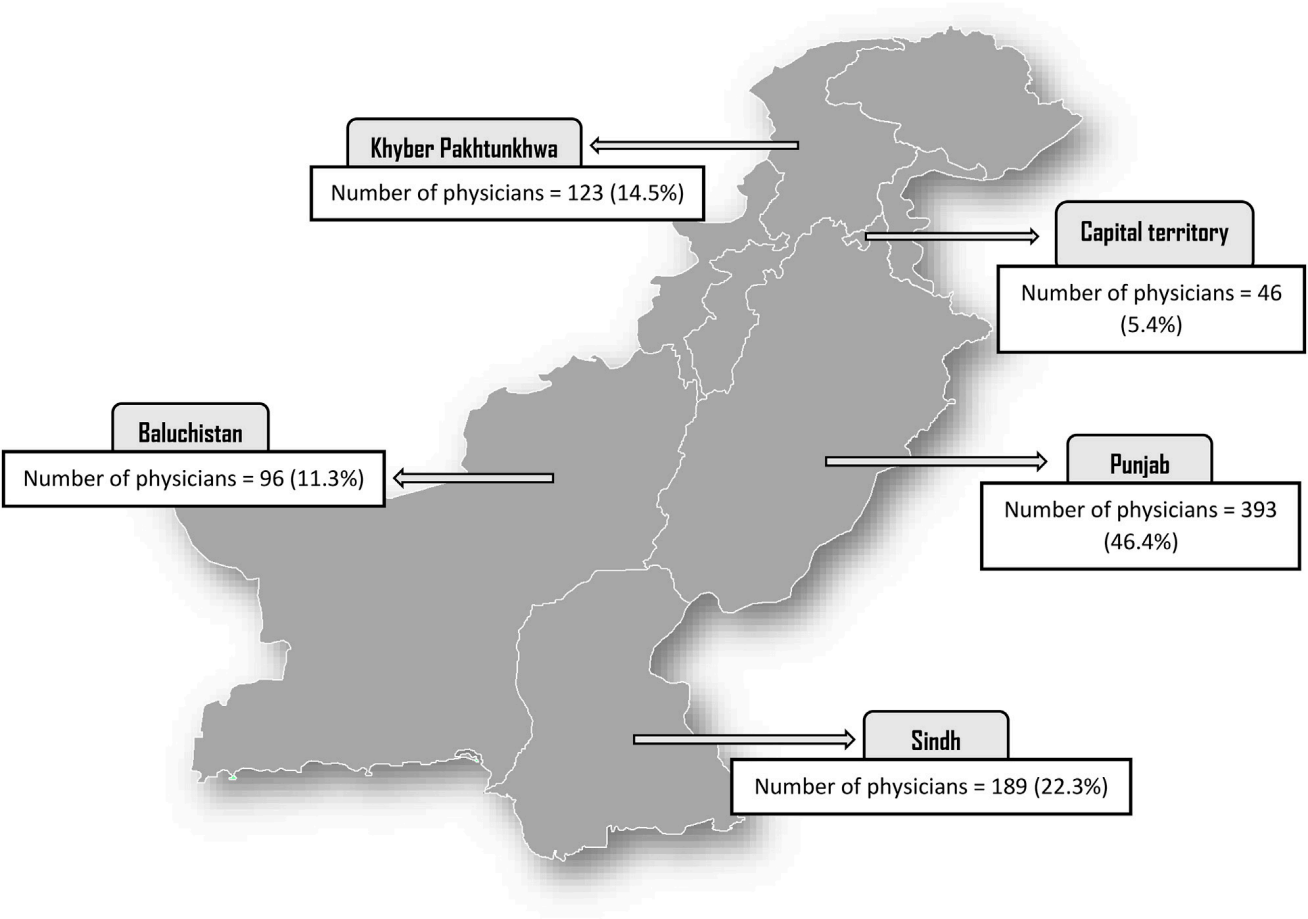
Map showing the sample distribution.

As shown in Figure 1, a total of 847 physicians [Punjab 46.4%, Sindh 22.3%, KPK 14.5%, Baluchistan 11.3%, and Islamabad (Capital territory) 5.4%] from across Pakistan completed the questionnaire (participation rate 82.9%). Demographic characteristics of the study participants are presented in Table 1. The majority of the participants were men (62.2%) belonging to 25–30 years age group (75.2%) followed by the 31–35 years age group (13.7%). Nearly half of the study participants had 2–5 years working experience, while 7.9% had more than 5 years working experience. As far as the education of the study participants is concerned, 80% were Bachelor of Medicine, Bachelor of Surgery (MBBS), while 20% of the physicians were Doctor of Medicine (MD)-Master of Surgery (MS)/Fellow of College of Physicians and Surgeons (FCPS). The majority of the study population (58.6%) were working in tertiary care/teaching hospitals, followed by 30.2% from secondary care hospitals. With regard to the working departments, the majority of participants (25.6%) were currently serving in emergency departments followed by surgery and allied departments (19.1%).

**TABLE 1:** Demographic information of participants (*n* = 847).

Variable	Frequency (*n*)	Percentage (%)
Gender		
Male	527	62.2
Female	320	37.8
Age (years)		
<25 years	56	6.6
25–30 years	637	75.2
31–35 years	116	13.7
≥36 years	38	4.5
Total experience		
≤1 year	349	41.2
>1–5 years	431	50.9
>5 years	67	7.9
Education		
MBBS	678	80.0
MD-MS/FCPS	169	20.0
Location		
Punjab	393	46.4
KPK	123	14.5
Baluchistan	96	11.3
Sindh	189	22.3
Islamabad	46	5.4
Hospital type		
Tertiary care	496	58.6
Secondary care	256	30.2
Primary care	95	11.2
Hospital department		
Emergency	217	25.6
Surgery and allied	162	19.1
Medicine and allied	144	17.0
Pediatric	104	12.3
ICU/CCU	114	13.5
Obstetrics and gynecology	50	5.9
Others	56	6.6

FCPS, Fellow of College of Physicians and Surgeons; HAMs, high alert medications; KPK, Khyber Pakhtunkhwa; MD, Doctor of Medicine; MS, Master of Surgery.

### 3.2 Awareness of High Alert Medications Administration

As documented in Table 2, the majority of the physicians (74.3%) provided correct responses concerning the administration of epinephrine in mild allergic reactions. About 66.7% of the physicians gave correct response about the administration of calcium chloride (CaCl_2_), and 71.3% knew that calcium gluconate and CaCl_2_ were not the same medicines and, therefore, not interchangeable. Surprisingly, only 57.3% gave correct response about the dose expression of insulin. Furthermore, only 63.8% and 55.3% of physicians correctly answered the questions related to administration of 15% KCl and 3% sodium chloride (NaCl). The median (IQR) score related to HAMs administration was 5 (3). As shown in Table 3, there was a significant difference of knowledge score related to HAMs administration (*p* < 0.05) between age, education (FCPS/MD > MBBS), experience, hospital, and province categories. Inter-group comparison of knowledge scores related to HAMs administration are shown in Table 4. The higher age group was found to be associated with better HAMs administration knowledge scores. Furthermore, physicians having >5 years work experience had significantly better knowledge scores compared with those with 1–5 and <1 year work experience.

**TABLE 2:** Awareness of high alert medications administration.

Questions	Correct response
	N (%)
For a patient who has mild allergic reaction, can we administer epinephrine (1 mg/ml) via fast intravenous push?	629 (74.3)
When an emergency happens, can we give 10% calcium chloride (10 ml) as a fast intravenous push (in 1–2 min)?	565 (66.7)
10% calcium gluconate and 10% calcium chloride are the same drugs and interchangeable	604 (71.3)
Dosage expression for insulin is either “ml” or “cc”	485 (57.3)
For chemotherapy dose calculation, body weight for children and body surface area for adult are considered	325 (38.4)
When an emergency such as ventricular fibrillation happens, can we administer 15% potassium chloride (10 ml) via fast IV push?	540 (63.8)
We can add 15% potassium chloride in Ringer's solution for rapid infusion	397 (46.9)
Insulin syringe can be replaced by 1 ml syringe	396 (46.8)
Fast intravenous infusion of 3% NaCl (500 ml) for patient who has low sodium level	468 (55.3)

**TABLE 3:** Comparison of high alert medications knowledge and practices among demographics using Mann–Whitney U and Kruskal–Wallis H tests.

Variable	Knowledge score				Practices score	
	HAM administration		HAM regulations			
	Mean rank	*p*-value	Mean rank	*p*-value	Mean rank	*p*-value
Gender						
Male	433.83	0.095	417.71	0.414	414.10	0.172
Female	405.24		431.68		437.60	
Age (years)						
<25 years	404.67	0.009	311.36	<0.001	391.26	0.514
25–30 years	412.85		413.86		422.53	
31–35 years	449.41		484.34		425.73	
≥36 years	538.86		550.39		468.50	
Total-experience						
≤1 year	372.01	<0.001	388.82	<0.001	409.11	0.007
>1–5 years	448.44		435.43		422.43	
>5 years	537.60		533.73		511.69	
Education						
MBBS	411.77	0.007	404.96	<0.001	413.80	0.028
MD-MS/FCPS	467.90		495.16		459.78	
Location						
Punjab	432.06	<0.001	452.81	<0.001	473.94	<0.001
KPK	358.98		449.19		408.23	
Baluchistan	461.62		201.06		296.99	
Sindh	401.73		444.93		362.05	
Islamabad	523.01		467.05		537.60	
Hospital type**						
Tertiary care	411.15	0.040	432.17	0.006	426.69	0.345
Secondary care	454.51		432.77		428.49	
Primary care	399.62		348.12		388.64	

FCPS, fellow of college of physicians and surgeons; HAMs, high alert medications; KPK, Khyber Pakhtunkhwa; MD, Doctor of Medicine; MS, Master of Surgery.

**TABLE 4:** Inter-group comparisons of high alert medications knowledge and practices score among selected demographic variables

Multiple comparisons	Knowledge score				Practice score	
	HAMs administration		HAMs regulation			
	Mean rank	*p*-value	Mean rank	*p*-value	Mean rank	*p*-value
Age						
<25 years *vs*. 25–30 years	344.31 *vs*. 347.24	0.916	268.44 *vs*. 353.91	**0.002**	--	--
<25 years *vs*. 31–35 years	81.04 *vs*. 89.13	0.314	62.13 *vs*. 98.27	**<0.001**	--	--
<25 years *vs*. ≥36 years	41.91 *vs*. 55.74	**0.015**	36.28 *vs*. 64.04	**<0.001**	--	--
25–30 years *vs*. 31–35 years	371.98 *vs*. 404.57	0.133	367.31 *vs*. 430.19	**0.004**	--	--
25–30 years *vs*. ≥36 years	332.25 *vs*. 434.34	**0.002**	331.85 *vs*. 441.14	**0.001**	--	--
31–35 years *vs*. ≥36 years	73.72 *vs*. 89.03	0.063	74.66 *vs*. 86.16	0.161	--	--
Total-experience						
≤1 year *vs*. >1–5 years	351.83 *vs*. 421.81	**<0.001**	366.72 *vs*. 409.76	**0.007**	383.65 *vs*. 396.05	0.442
≤1 year *vs*. >5 years	195.18 *vs*. 277.86	**<0.001**	197.10 *vs*. 267.89	**<0.001**	200.46 *vs*. 250.37	**0.002**
1–5 years *vs*. >5 years	242.62 *vs*. 293.74	**0.006**	241.67 *vs*. 299.84	**0.002**	242.38 *vs*. 295.32	**0.005**
Location						
Punjab *vs*. KPK	269.15 *vs*. 224.47	**0.003**	259.17 *vs*. 256.37	0.854	269.21 *vs*. 224.28	**0.003**
Punjab *vs*. Baluchistan	241.45 *vs*. 259.52	0.256	273.63 *vs*. 127.81	**<0.001**	264.38 *vs*. 165.65	**<0.001**
Punjab *vs*. Sindh	298.37 *vs*. 277.22	0.151	293.42 *vs*. 287.50	0.687	316.16 *vs*. 240.21	**<0.001**
Punjab *vs*. Islamabad	215.04 *vs*. 262.34	**0.016**	219.33 *vs*. 225.70	0.744	216.87 *vs*. 246.78	0.127
KPK *vs*. Baluchistan	97.82 *vs*. 125.60	**0.001**	136.87 *vs*. 75.58	**<0.001**	123.72 *vs*. 92.42	**<0.001**
KPK *vs*. Sindh	145.83 *vs*. 163.45	0.087	156.98 *vs*. 156.19	0.938	168.28 *vs*. 148.84	0.062
KPK *vs*. Islamabad	76.65 *vs*. 107.34	**<0.001**	84.17 *vs*. 87.23	0.714	76.35 *vs*. 107.65	**<0.001**
Baluchistan *vs*. Sindh	157.39 *vs*. 135.69	**0.033**	87.39 *vs*. 171.25	**<0.001**	127.24 *vs*. 151.00	**0.021**
Baluchistan *vs*. Islamabad	67.78 *vs*. 79.26	0.116	56.27 *vs*. 103.28	**<0.001**	58.63 *vs*. 98.37	**<0.001**
Sindh *vs*. Islamabad	111.23 *vs*. 145.80	**0.002**	116.74 *vs*. 123.17	0.559	108.46 *vs*. 157.18	**<0.001**
Hospital type						
Tertiary *vs*. secondary care	363.18 *vs*. 402.32	**0.018**	376.30 *vs*. 376.89	0.972	--	--
Tertiary *vs*. primary	297.02 *vs*. 290.65	0.736	305.44 *vs*. 246.70	**0.002**	--	--
Secondary *vs*. primary	181.69 *vs*. 160.66	0.081	186.08 *vs*. 148.84	**0.002**	--	--

HAMs, high alert medications; KPK, Khyber Pakhtunkhwa. Bold values indicates that *p* < 0.05.

### 3.3 Awareness of High Alert Medications Regulations

As shown in Table 5, approximately two-thirds of our study participants knew “mg” and “gram” should not be replaced with “ampule” or “vial” for dose expression. Moreover, approximately half of the participants knew not to use inconsistent abbreviations (“U” or “IU” instead of “units”) for the dose expression of HAMs. Only 48.3% of study participants knew insulin and heparin should not be stored together. Additionally, 53.5% provided correct answers regarding the storage of atracurium. The median (IQR) knowledge score related to HAMs regulation was 5 (2). As shown in Table 3, there was a significant difference of knowledge score related to HAMs regulation between age, experience, education (FCPS/MD > MBBS), and province/location categories of physicians. Inter-group comparisons (*post hoc* analysis) of knowledge score related to HAMs regulations are shown in Table 4. Increasing age was significantly associated with better HAMs regulation knowledge scores. Physicians having >5 years work experience had significantly better knowledge about HAMs regulations than those with 1–5 and <1 year work experience. Furthermore, physicians working in tertiary and secondary care settings had better scores than those from primary care settings.

**TABLE 5:** Awareness of high alert medications regulations.

Questions	Correct response
	N (%)
It is right to use “ampule” or “vial” for dose expression instead of “mg” or “gm”?	563 (66.5)
Distinctive labeling should be given to all high alert medications that look similar	666 (78.6)
We can use “U” instead of unit for dose expression of high alert medications	424 (50.1)
For convenience, heparin and insulin can be stored together	409 (48.3)
High alert medications should have multiple concentrations for health professionals to choose	252 (29.8)
If patient can tolerate, potassium chloride can be administered orally instead of intravenous route	400 (47.2)
Since 15% potassium chloride is frequently used, it should be easily and freely accessed	454 (53.6)
For pediatric dose, use teaspoon for dose expression	272 (32.1)
Fentanyl skin patch as regulated narcotic	340 (40.1)
If a ward stores atracurium for tracheal intubation, the drug should be stored with other drugs for quick accessibility	453 (53.5)

### 3.4 Overall Knowledge Related to High Alert Medications

**FIGURE 2:**
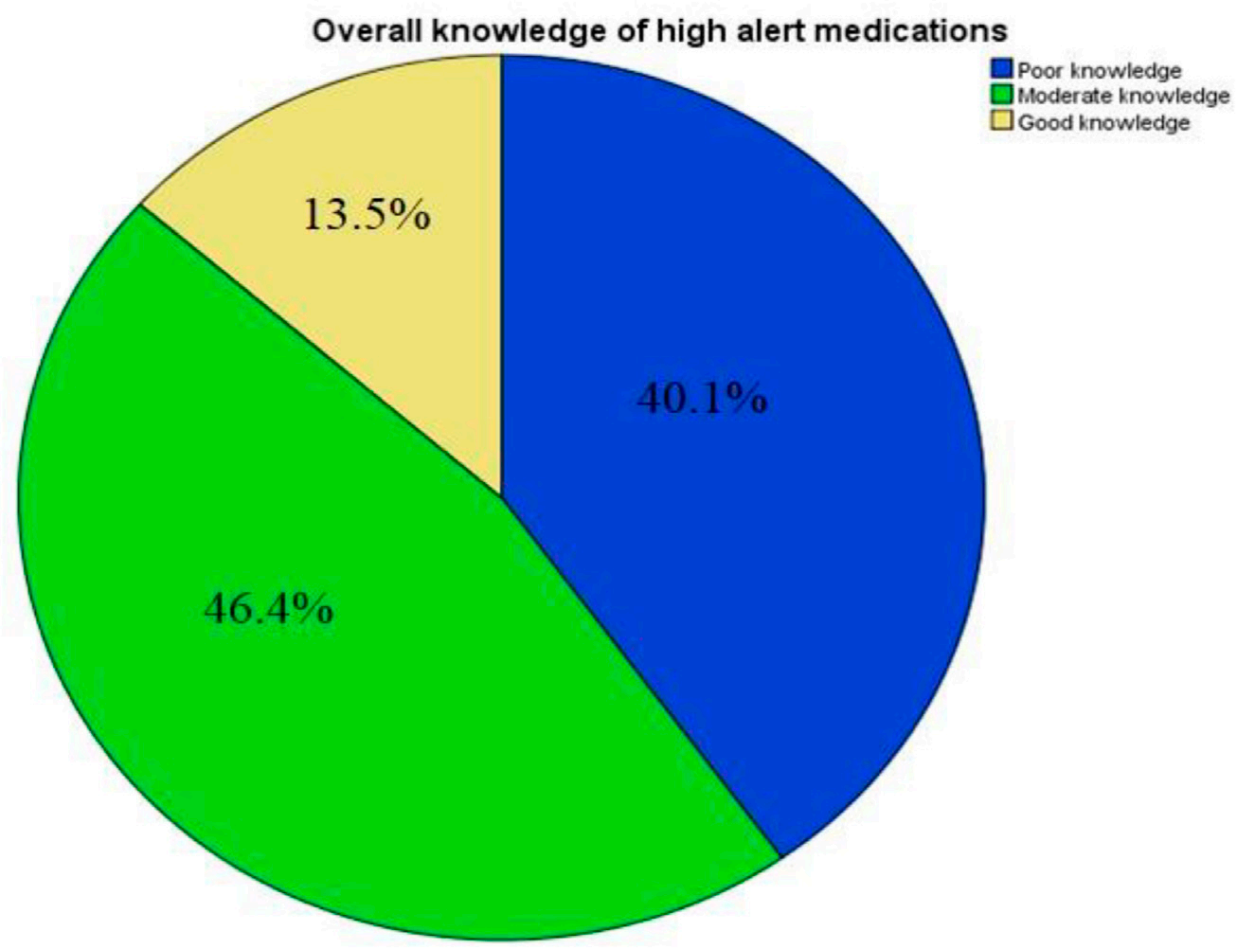
Overall high alert medications-related knowledge of Pakistani physicians.

As depicted in Figure 2, majority (46.4%) of physicians had moderate/average knowledge about HAMs. Only 13.5% of the respondents were found to possess excellent knowledge (score ≥70%) about HAMs.

### 3.5 Practices Related to High Alert Medications

As shown in Table 6, only 14.4% of the physicians reported of never delivering verbal orders about HAMs to the nursing staff, hence following good practices related to the HAMs. Moreover, 55.3% and 55.8% reported of following standard operating procedures on HAMs in their hospitals and prescribing these drugs in a designated place in the patients' medical record, respectively. About 65% of the physicians reported they prescribe HAMs with complete information to nursing staff to avoid errors. Furthermore, 53% always instructed nursing staff to “double-check” prior to administration. The median HAMs practice score was 20 (4), with the majority (57.9%) of study participants following adequate practices (score >70%) related to HAMs. Total experience, location, and hospital type of the study participants had significant associations (*p* < 0.05) with median score towards HAM practices (Table 3). Those having a FCPS or MD qualification had significantly better practices than those having only a MBBS qualification. Moreover, as shown in Table 4, physicians having >5 years work experience had significantly better practices than those with 1–5 and <1 year work experience. In addition, physicians from the hospitals in Islamabad and Punjab had better practices than in the others (Table 4).

**TABLE 6:** Practices of high alert medications regulations.

Question	N (%)				
	Always	Often	Sometimes	Rarely	Never
I deliver verbal orders to nursing staff for administration of high alert medications to my patients in case of emergency	217 (25.6)	194 (22.9)	191 (22.6)	123 (14.5)	122 (14.4)
I write prescription orders for high alert medications in designated place in patient's medical file	473 (55.8)	191 (22.6)	95 (11.2)	47 (5.5)	41 (4.8)
I instruct nursing staff to “double check” before administration of high alert medications to my patients	447 (52.8)	178 (21.0)	111 (13.1)	65 (7.7)	46 (5.4)
I follow the standard operating procedures for administrating of high alert medications	468 (55.3)	210 (24.8)	109 (12.9)	32 (3.8)	28 (3.3)
I prescribe high alert medications with complete instructions for nursing staff about their administration to avoid any error	547 (64.6)	163 (19.2)	84 (9.9)	22 (2.6)	31 (3.7)

## 4 Discussion

We believe this is the first study of its kind in Pakistan to evaluate the knowledge and practices on HAMs-related administration and regulations among physicians throughout Pakistan, building on our previous study among nurses (Salman et al., 2020). There were areas of encouragement. However, we identified a number of areas of concern that need to be addressed. Epinephrine is recommended as first-line therapy in anaphylaxis, and it should be administered at a dose of 0.1 mg in the diluted form via slow infusion (in 5–15 min). However, one-fourth of the Pakistani physicians in our study were unable to administer this HAM according to these guidelines. Our previous study conducted among registered nurses revealed similar findings about the administration of epinephrine (Salman et al., 2020). Our findings though are in line with a recent study reported by Wijekoon et al. (2021) from Sri Lanka who revealed that majority of the study population knew that epinephrine is the first-line drug to treat anaphylaxis along with correct dose in children and adults (Wijekoon et al., 2021). Encouragingly, our findings are better than previous studies reported from the United Kingdom, where 15% of physicians were familiar with the recommended way of epinephrine administration in anaphylaxis (Droste and Narayan, 2010; Droste and Narayan, 2012; Tyree et al., 2012).

Similarly, CaCl_2_, another HAM, indicated for electrolytes abnormalities to advance cardiac life support (Chakraborty and Can, 2021), carries the potential of cutaneous burning sensation and peripheral vasodilation when administered via intravenous (IV) push. Consequently, it is recommended to administer CaCl_2_ slowly (in 5–10 min) and repeat the same if necessary (Lindner et al., 2020). The concentration of calcium in CaCl_2_ is three times than that present in calcium gluconate, and hence they cannot be replaced by each other. However, one-third of physicians in our study were not aware of the right way of CaCl_2_ administration and even considered that CaCl_2_ can be replaced with calcium gluconate. These findings though are in line with the previous study from the United Kingdom (Powell et al., 2013) that reported poor knowledge among doctors about the IV administration of different IV fluids including CaCl_2_.

Abrupt administration of concentrated KCl carries the risk of arrhythmias, cardiac arrest, and even deaths reported throughout the world (Grissinger, 2011; Bertol, et al., 2012). Consequently, physicians must prescribe KCl in a diluted form and administer it slowly to avoid complications. However, one-third of our study population were not aware of the right way of KCl administration, and less than half of the participants agreed to add KCl in Ringer's solution despite the chances of metabolic alkalosis. These findings though are similar to the results of a previous study from Japan that reported inadequate physicians' knowledge about the KCl administration necessitating interventions for improvement (Nakatani et al., 2019). Another HAM, insulin, needs to be administered carefully with the right dose usually calculated in international units. Moreover, it should be administered using a dedicated insulin syringe. However, of concern is that nearly half of the physicians in our study were unaware that dose expression for insulin is in international units and not in “ml.” Our findings are contrary though to those of Bain et al. (2019) who reported a better understanding of insulin-related factors among junior doctors, pharmacists, and nurses from the United Kingdom where more than two-thirds of consultant physicians possessed good knowledge about insulin and its administration (Bain et al., 2019).

Incorrect dose calculation, especially among children, is one of the major contributing factors in the emergence of adverse drug events (ADEs). Consequently, physicians are expected to be precise while prescribing to them. However, only one-third of physicians in our study gave correct responses to the question about dose calculation in children and adults. These findings are contrary to the result of a previous study from Australia that reported a high percentage of medical students were well aware of the dose calculation methods (Harries and Botha, 2013). Our survey revealed that the age, level of education, working experience, and hospital type are related to better HAMs administration scores among physicians. These findings are in line with a recent study reported from KPK Province, Pakistan, where age and working experience of the medical doctors are associated with better drug safety knowledge (Sharif et al., 2021). In our previous study conducted among nurses, no associations though were observed between HAMs administration awareness with age, experience, and level of education (Salman et al., 2020). We are not sure of the reasons why; however, this may relate to greater learning curves among physicians as they gain experience. We also found that physicians currently serving in tertiary care/teaching hospitals possessed better HAMs awareness compared with primary and secondary hospital physicians, similar to the previous study conducted among nurses from tertiary care hospitals that found they also had better HAMs knowledge (Salman et al., 2020). This may reflect a greater complexity of cases in tertiary hospitals and the need to be more vigilant regarding HAMs.

HAMs typically require special precautions during their prescription, administration, and storage in the wards. Consequently, special regulations are typically needed to avoid any harm due to their inappropriate use. Using inconsistent abbreviations while prescribing HAMs carries an even greater risk of MEs, so prescribers need to be careful while prescribing them. The use of only “U” instead of “units” needs to be avoided as an appropriate strategy for the prescriber, as discussed in the previous study (Zyoud et al., 2019). Insulin is a refrigerated medicine (2–8°C), whereas heparin should be stored at room temperature under lock and key (Riley and Meyers, 2016). Regarding atracurium regulation, it should be refrigerated at 2–8°C to preserve potency, and it should be used within 14 days, even if re-refrigerated. This is a concern as an earlier study by Engels et al. revealed that 6% of HAMs-related errors were associated with their inappropriate storage (Engels and Ciarkowski, 2015). Again, some physicians in our study were not familiar with these regulations on HAMs. This is different from our findings among registered nurses where the majority of study participants were familiar with HAM related regulations (Salman et al., 2020). This may be because nurses typically administer heparin and consequently are expected to know more about storage requirements.

Verbal orders about medication are associated with MEs due to poor audition, understanding, and wrong word heard; thus, verbal orders about HAMs should not be delivered by the physicians (Moghaddasi et al., 2017). For the safety of patients, there is a recommended place in the patient's medical record for the prescription of HAMs in order that all members of the health care provision team can easily identify it and be attentive while dealing with that drug. After prescribing, physicians must instruct the nursing staff to use a “double-check policy” (Hewitt et al., 2016) to confirm the patient's name, drug name, dose, frequency, and route of administration before initiation of medication administration. Encouragingly, most of our study physicians followed good practices including prescribing HAMs with complete instructions, stressing double-check policies, and following standard operating procedures developed to reduce HAM-related errors as all these strategies have been devised to reduce errors related to HAMs.

### 4.1 Strengths and Limitations

This was the first study of its kind to evaluate the knowledge and practices on HAMs-related administration and regulations among physicians throughout Pakistan. We believe our comprehensive study including an appreciably higher number of physicians than the sample size calculations provides policy makers in Pakistan with specific guidance on future measures to enhance patient safety in hospitals. However, we are aware of limitations that were inherent in our study. Firstly, due to COVID-19 restrictions, investigators were unable to approach working physicians directly. Consequently, an online cross-sectional study was conducted using convenient sampling. We are aware though that this sampling method is associated with some shortcomings, e.g., sampling bias, over-representation, and non-generalizability. Secondly, most of the study participants recruited in our survey were MBBS degree holders with comparatively less working experience which may limit the generalizability of the study. Furthermore, as the physicians working in private sector hospitals were not included in our study, our findings may not be representative of the entire Pakistani physicians' population. However, we believe that despite these limitations, this study provides much needed baseline information related to HAMs knowledge among Pakistani physicians to provide future direction to key policy makers and others in Pakistan.

## 5 Conclusion and Recommendations

The current survey showed that most of the Pakistani physicians possessed average knowledge about the administration and regulation of HAMs. Similar trends were observed about their practices related to HAM during their routine work. Consequently, policy makers and others need to instigate future measures to enhance patient safety during routine health care provision. All concerned authorities should take appropriate steps to revise the current curriculum in medical institutions to ensure key subjects about patient's safety, in particular, HAMs, are included. All hospital settings should also ensure continuous trainings/workshops for the physicians with a special focus on HAMs and patient safety. Standard operating procedures related to HAMS administration and regulations should also be introduced and monitored at the institutional level. Moreover, hospitals should seek to establish multidisciplinary health care provision teams, if not already implemented, through engaging board-certified clinical pharmacists and specialized nurses to develop guidelines and standard operating procedures to minimize future harms related to the medication use process, and continually monitor activities.

## Data Availability

The original contributions presented in the study are included in the article/supplementary material; further inquiries can be directed to the corresponding authors.
